# Effect of mixed meal replacement of soybean meal on growth performance, nutrient apparent digestibility, and gut microbiota of finishing pigs

**DOI:** 10.3389/fvets.2024.1321486

**Published:** 2024-02-01

**Authors:** Zhentao He, Shuai Liu, Xiaolu Wen, Shuting Cao, Xianliang Zhan, Lei Hou, Yaojie Li, Shaozhen Chen, Huayu Zheng, Dongyan Deng, Kaiguo Gao, Xuefen Yang, Zongyong Jiang, Li Wang

**Affiliations:** State Key Laboratory of Livestock and Poultry Breeding; Key Laboratory of Animal Nutrition and Feed Science in South China, Ministry of Agriculture and Rural Affairs; Guangdong Provincial Key Laboratory of Animal Breeding and Nutrition; Maoming Branch, Guangdong Laboratory for Lingnan Modern Agriculture, Institute of Animal Science, Guangdong Academy of Agricultural Sciences, Guangzhou, China

**Keywords:** rapeseed meal, cotton meal, sunflower meal, finishing pigs, short-chain fatty acid

## Abstract

**Introduction:**

This study was carried out to investigate the effects of mixed meal (rapeseed meal, cotton meal, and sunflower meal) replacement soybean meal on growth performance, nutrient apparent digestibility, serum inflammatory factors and immunoglobulins, serum biochemical parameters, intestinal permeability, short-chain fatty acid content, and gut microbiota of finishing pigs.

**Methods:**

A total of 54 pigs with an average initial weight of 97.60 ± 0.30 kg were selected and randomly divided into 3 groups according to their initial weight, with 6 replicates in each group and 3 pigs in each replicate. The trial period was 26 days. The groups were as follows: control group (CON), fed corn-soybean meal type basal diet; Corn-soybean-mixed meal group (CSM), fed corn-soybean meal-mixed meal diet with a ratio of rapeseed meal, cotton meal, and sunflower meal of 1:1:1 to replace 9.06% soybean meal in the basal diet; Corn-mixed meal group (CMM), fed a corn-mixed meal diet with a ratio of Rapeseed meal, Cotton meal and Sunflower meal of 1:1:1 to replace soybean meal in the basal diet completely. The crude protein level of the three diets was maintained at 12.5%.

**Results:**

Our findings revealed no significant impact of replacing soybean meal with the mixed meal (rapeseed meal, cotton meal, and sunflower meal) on the ADG (Average daily gain), ADFI (Average daily feed intake), and F/G (Feed gain ratio) (*P* > 0.05), or crude protein, crude fat, and gross energy (*P* > 0.05) in the diet of finishing pigs. Compared with the CON group, the serum interleukin 6 (IL-6) and interleukin 10 (IL-10) concentrations were significantly decreased in the CMM group (*P* < 0.05). However, there is no significant effect of the mixed meal (rapeseed meal, cotton meal, and sunflower meal) replacing soybean meal in the diet on the serum interleukin 1β (IL-1β), interleukin 8 (IL-8), tumor necrosis factor-alpha (TNF-α), immunoglobulin A (IgA), immunoglobulin G (IgG), and immunoglobulin M (IgM) concentrations (*P* > 0.05). Concordantly, there is no significant effect of mixed meal (rapeseed meal, cotton meal, and sunflower meal) replacing soybean meal in the diet on the serum antioxidant capacity, such as total antioxidant capacity (T-AOC), catalase (CAT), and malondialdehyde (MDA) levels of finishing pigs. Moreover, compared with the CON group, serum low-density lipoprotein (LDL-C) levels were significantly lower in the CSM group (*P* < 0.05) and their total bilirubin (TBIL) levels were significantly lower in the CMM group (*P* < 0.05). There is not a significant effect on serum D-lactate and diamine oxidase (DAO) concentrations (*P* > 0.05). The next section of the survey showed that the replacement of soybean meal with a mixed meal (rapeseed meal, cotton meal, and sunflower meal) in the diet did not significantly influence the acetic acid, propionic acid, butyric acid, valeric acid, isobutyric acid, and isovaleric acid in the colon contents (*P* > 0.05). Furthermore, compared with the CON group, the CMM group diet significantly increased the abundance of Actinobacteria at the phylum level (*P* < 0.05), *U_Actinobacteria* at the class level (*P* < 0.05), and *U_Bacteria* at the class level (*P* < 0.05). The result also showed that the CMM group significantly reduced the abundance of *Oscillospirales* at the order level (*P* < 0.05) and *Streptococcaceae* at the family level (*P* < 0.05) compared with the CON group. The Spearman correlation analysis depicted a statistically significant positive correlation identified at the class level between the relative abundance of *U_Bacteria* and the serum T. BILI concentrations (*P* < 0.05). Moreover, a significant negative correlation was detected at the order level between the relative abundance of *Oscillospirales* and the levels of acetic and propionic acids in the colonic contents (*P* < 0.05). Additionally, there was a significant positive correlation between the serum concentrations of IL-6 and IL-10 and the relative abundance of the family *Streptococcaceae* (*P* < 0.05).

**Discussion:**

This study demonstrated that the mixed meal (rapeseed meal, cotton meal, and sunflower meal) as a substitute for soybean meal in the diet had no significant negative effects on the growth performance, nutrient apparent digestibility, serum immunoglobulins, serum antioxidant capacity, intestinal permeability, short-chain fatty acid content, and diversity of gut microbiota of finishing pigs. These results can help develop further mixed meals (rapeseed meal, cotton meal, and sunflower meal) as a functional alternative feed ingredient for soybean meals in pig diets.

## 1 Introduction

Soybean meal is the main protein source for non-ruminants due to its substantial protein content, ranging from 44% to 49%. This high protein value is essential for supplying an adequate amount of amino acids required for optimal animal growth and development (1, 2). Predominantly, soybean meal serves as the primary protein source in swine feeds across the globe. However, the swine industry's heavy reliance on the importation of significant quantities of soybean meal has sparked growing concerns. To maintain a competitive stance in the global marketplace, the swine industry is proactively seeking sustainable and feasible strategies. These strategies aim to generate and supply nutritional energy and minerals, while simultaneously ensuring appropriate yield and maintaining optimum animal performance levels. As the global demand for livestock and poultry production continues to escalate, identifying alternative raw materials that can replace soybean meal has become increasingly critical.

Alternative native protein feedstocks are required to increase pig production's sustainability and self-sufficiency. Rapeseed meal, cottonseed meal, and sunflower seed meal are typical by-products of agricultural production in China. These by-products exhibit favorable characteristics such as higher protein content, larger yield, and lower cost. As plant-based feed protein raw materials, they offer promising potential to replace soybean meal in pig production. Previous studies have shown that the scientific and rational application of this type of protein raw materials can effectively reduce the amount of soybean meal in balanced feed, which has a positive significance in alleviating the dependence on soybean meal (3, 4).

There are differences in the use of rapeseed meal, cottonseed meal, and sunflower meal in the growing swine stage of swine production. Previous studies have highlighted the potential benefits of incorporating rapeseed meal and fava beans in the diets of growing pigs (108.7 ± 4.2 kg final body weight). Specifically, these additions have been shown to improve feed conversion rates during the finishing period and increase the concentration of free amino acids in the blood (5). Cottonseed meal exhibits similar crude protein, mineral, and vitamin content to soybean meal. Research has indicated that the partial replacement of 2.5% soybean meal with cottonseed meal in the diet of growing pigs (13.18 to 39.81 kg) does not significantly impact growth performance (4). Furthermore, Kim et al. (6) have observed that growing pigs (initial body weight of 19.3 ± 1.8 kg) demonstrate enhanced digestive utilization efficiency when fed high-protein sunflower seed meal. All of the above studies show that mixed meals (rapeseed meal, cotton meal, and sunflower meal) are a promising protein material for swine.

However, it remains unclear whether the replacement of soybean meal in finishing pigs' diets with a mixture of rapeseed meal, cottonseed meal, and sunflower meal is associated with changes in growth performance and intestinal microbial diversity in finishing pigs. Therefore, the purpose of this experiment was to investigate the effects of using miscellaneous meal in place of soybean meal in diets on growth performance, nutrient apparent digestibility, serum immunoglobulins, serum antioxidant capacity, intestinal permeability, short-chain fatty acid content, and diversity of gut microbiota of finishing pigs.

## 2 Materials and methods

### 2.1 Animals

The animal treatment protocols implemented in this study were reviewed and approved by the Animal Care and Use Committee of the Guangdong Academy of Agricultural Sciences. The protocols adhered to the guidelines outlined in the Guide for the Care and Use of Animals for Research and Teaching (Authorization No. GAIASIAS-2022-022).

A total of 54 pigs (27 barrows and 27 gilts, 150 days) with an average initial weight of 97.60 ± 0.30 kg were selected and randomly divided into 3 treatment groups according to their initial weight. Each treatment will encompass 6 replicates with 3 pigs per pen (9 barrows and 9 gilts). The finishing pigs utilized in this study are sourced from the breeding test base of the Institute of Animal Science, Guangdong Academy of Agricultural Sciences. The trial period was 26 days. The groups were as follows: control group (CON), fed corn-soybean meal type basal diet; Corn-soybean-mixed meal group (CSM), fed corn-soybean meal-mixed meal diet with a ratio of Rapeseed meal, Cotton meal, and Sunflower meal of 1:1:1 to replace 9.06% soybean meal in the basal diet; Corn-mixed meal group (CMM), fed a corn-mixed meal diet with a ratio of rapeseed meal, cotton meal and sunflower meal of 1:1:1 to replace soybean meal in the basal diet completely. All 18 pens were identical, with the same covered area (3 m^2^/pig), and were equipped with similar troughs for feed concentrates and water. Pigs were provided *ad libitum* access to water and feed during the entire experimental trial. At the beginning and the end of the trial, the trial pigs were weighed after 12 h of fasting.

### 2.2 Experimental diets

The nutritional regimes for the experimental diets were devised with calculated precision, adhering stringently to the specifications outlined by the National Research Council (NRC, 2012, p. 248–249) for finishing pigs. These guidelines encompass both the requisite nutrient levels for optimal growth and the nutrient profiles of a variety of feedstuffs. The experimental formulations were rendered isonitrogenous, with a standardized crude protein content of 12.5% across all diets. Moreover, the dietary compositions were finely tuned to maintain homogeneity in the levels of essential nutrients, including metabolizable energy, calcium, standardized total tract digestible phosphorus (STTD P), and standardized ileal digestible lysine. The concentration of these nutrients was controlled within a tight tolerance of ±0.6%, thereby ensuring that the nutritional integrity was consistent among the different treatment groups and mitigating any potential variances that could impact the experimental outcomes.

To enhance the precision of fecal matter identification and subsequent nutrient digestibility evaluation, 0.4% titanium dioxide was incorporated as a non-absorbable marker within the feed matrix. The feed was subsequently pelletized to promote consistent intake across all subjects and to reduce the potential for feed wastage.

Comprehensive details regarding the composition and proportions of ingredients in the basal diet are systematically cataloged in Table 1. This methodological rigor was paramount to ensure that the dietary interventions were tailored to the physiological demands of the animals throughout the finishing phase, thereby enabling a robust and reliable evaluation of their growth performance and overall feed conversion efficiency.

**Table 1:** Composition and nutrient level of the experimental diets (air-dried basis, %).

**Ingredients, %**	**Treatments** ^**a**^		
	**CON**	**CSM**	**CMM**
Corn	56.05	52.94	50.15
Cassava	25.00	25.00	25.00
Soybean meal	16.46	7.40	
Rapeseed meal		3.52	6.46
Cotton meal		3.52	6.46
Sunflower meal		3.52	6.46
Soybean oil		1.50	2.75
Calcium carbonate	0.60	0.60	0.58
Calcium phosphate	0.09	0.04	0.03
NaCl	0.40	0.40	0.40
L-lysine hydrochloride	0.08	0.21	0.33
L-Threonine	0.01	0.04	0.06
L-Tryptophan			0.01
Choline chloride	0.10	0.10	0.10
Premix^b^	1.21	1.21	1.21
Total	100.00	100.00	100.00
**Nutrient level**			
Metabolic energy, MJ/kg	13.86	13.80	13.75
Crude protein	12.50	12.50	12.50
Ca	0.49	0.49	0.49
STTD P	0.22	0.22	0.22
SID Lys	0.61	0.61	0.61
SID Met	0.18	0.19	0.20
SID Met+Cys	0.38	0.39	0.40
SID Thr	0.40	0.40	0.40
SID Trp	0.13	0.11	0.11

^a^CON, the control group was fed corn-soybean meal basal diet; CSM, the corn-soybean meal mixed meal group was fed with mixed meal to partially replace soybean meal in the basal diet; CMM, the corn mixed meal group was fed with mixed meal as a complete replacement of soybean meal in the basal diet. ^b^The premix provided the following per kg of diets: VA 4 500 IU, VD2 100 IU, VE 22.5 mg, VK 3.75 mg, VB1 2.25 mg, VB2 7.5 mg, nicotinic acid 30 mg, D-pantothenic acid 11.25 mg, folic acid 0.75 mg,VB6 3 mg, VB12 0.03 mg, biotin 0.08 mg, Fe(FeSO4•H2O) 112.5 mg, Cu(CuSO4•5H2O) 6 mg, Mn(MnSO4•H2O) 4.5 mg, Zn(ZnSO4•H2O) 60 mg, I(CaI2O6) 0.14 mg, Se(Na2SeO3) 0.3 mg.

### 2.3 Sample collection

After 26 days of experimental period, the pigs in each group were weighed. Three days before the end of the experiment, fresh fecal samples were collected from pigs before feeding in the morning for 3 consecutive days. Fresh, uncontaminated fecal samples were collected once a day at 7:00 a.m., weighed and mixed with 10% hydrochloric acid 10 mL per 100 g sample for nitrogen fixation treatment, and stored at −20°C for subsequent analysis. In addition, fresh unground fecal samples were received and stored at −80°C for gut microbial composition and diversity. At the conclusion of the experiment, one pig from each replicate, chosen based on average body weight, was selected for blood sampling. Following a fasting period of 12 h, 20 mL of blood was collected from the pig's ear vein. The collected blood was then centrifuged at a speed of 3,000 rpm for a duration of 10 min to yield the serum samples.

### 2.4 Growth performance

Feed consumption of each replicate was recorded from the beginning to the end of the experiment. The average daily gain (ADG), average daily feed intake (ADFI), and feed-to-gain ratio (F/G) were calculated accordingly. The equations utilized for the determination of growth performance metrics are specified below:


$$\begin{array}{l} \text{ADG}=\text{(Final body weight}-\text{Initial body weight)/days of feeding;}\\ \text{ADFI}=\text{(total feed consumed}-\text{residual feed)/}\\ \;\text{(duration of feeding period}\times \text{number of pigs);}\\ \text{F/G}=\text{ADFI/ADG}. \end{array}$$

### 2.5 Nutrient apparent digestibility

All diets were given a starting dose of titanium dioxide (0.4%), which was used as an indigestible marker of nutrient apparent digestibility. In pens, feces were collected, dried, sampled, and stored at −20°C for analysis. All of the growing pigs' excrement was defrosted, blended, and then baked at 65°C for 72 h before the natural moisture was restored at room temperature for 24 h.

The estimation of crude protein content was performed by multiplying the total nitrogen content, which was measured using the Kjeltec 8400 analyzer (FOSS Analytical AB, Sweden), by a conversion factor of 6.25. Additionally, the determination of crude fat content was carried out using an automated extraction analyzer (XT 15i, Ankom Technology, USA).; the total energy content in the rations and manure samples The total energy content of the rations and manure samples was determined using an oxygen bomb calorimeter (6400, Parr Instrument, USA) according to the international standard ISO 9831:1998 method. The concentration of TiO2 in all samples (feed and fecal) was measured according to Myers et al. (7).

Nutrient apparent digestibility was calculated as follows (8): Apparent nutrient digestibility (%) = [1 – (TiO2 content in the dietary/TiO2 content in the fecal sample) × (nutrient content in the fecal sample/nutrient content in the dietary)] × 100.

### 2.6 Enzyme-linked immunosorbent assay

In this study, the detection of serum inflammatory factors and immunoglobulins was carried out using kits supplied by Jiangsu Meimian Industrial Co., Ltd. (Jiangsu, China). The determinations were made by the kit's provided instructions, including interleukin 1β (IL-1β, Item No.: MM-042201), interleukin 6 (IL-6, Item No.: MM-041801), interleukin 8 (IL-8, Item No.: MM-041701), interleukin 10 (IL-10, Item No.: MM-042501), tumor necrosis factor-alpha (TNF-α, Item No.: MM-038301), immunoglobulin A (IgA, Item No.: MM-090501), immunoglobulin G (IgG, Item No.: MM-040301), immunoglobulin IgM (IgM, Item No.: MM-040201), D-lactate (D-lactate, Item No.: MM-3373201), and diamine oxidase (DAO, Item No.: MM-043801).

### 2.7 Analyses of antioxidant capacity in the serum

The antioxidant indexes of serum samples including total antioxidant capacity (T-AOC), catalase (CAT), and malondialdehyde (MDA) were measured using the commercial kits from Nanjing Jiancheng Institute of Biological Engineering (Nanjing, China) according to the manufacturer's instructions.

### 2.8 Serum biochemical parameters measurement

A range of serum biochemical markers were evaluated in this study. This included serum total protein (TP), alanine aminotransferase (ALT), creatinine (CRE), aspartate aminotransferase (AST), alkaline phosphatase (ALP), albumin (ALB), urea (UREA), glucose (GLU), triglyceride (TG), total cholesterol (CHO), high-density lipoprotein (HDL-C), low-density lipoprotein (LDL-C), and total bilirubin (TBIL). These parameters were determined according to the methods described previously (9).

Additionally, porcine transferrin (TRF) and ceruloplasmin (CP) were identified using kits obtained from Nanjing Jiancheng Bioengineering Institute (Nanjing, China).

### 2.9 Measurement of short-chain fatty acid

Short-chain fatty acids (including acetic acid, propionic acid, butyric acid, valeric acid, isobutyric acid, and isovaleric acid) were quantified according to the methods described previously (9).

The experiment utilized 0.5 g of colon contents as the sample. To dissolve it completely, 1.5 mL of water was added and then centrifuged. Next, 1 mL of the resulting supernatant was combined with 200 μL of crotonic acid (concentration: 42 mmol/L) and 200 μL of metaphosphoric acid solution (concentration: 10%), ensuring thorough mixing. The mixture was then refrigerated at 4°C overnight. Subsequently, centrifugation was performed at 4°C for 10 min to obtain the supernatant, which was mixed with an equal proportion of ether. After allowing it to stand for 5 min, the ether layer was extracted using a disposable syringe and filtered through a 0.22 μm organic membrane. Finally, the filtered liquid was injected into a brown injection bottle.

### 2.10 Analysis of gut microbial composition and diversity

This study employed 16S rRNA sequencing to evaluate the impact of replacing a soybean meal with a mixed meal (rapeseed meal, cotton meal, and sunflower meal) on the colonic contents' flora of finishing pigs. Genomic DNA was extracted using the OMEGA Soil DNA Kit (No.: D562501; Omega Bio-Tek, Norcross, GA, USA) following the manufacturer's instructions. The quantity and quality of the extracted DNA were assessed using a NanoDrop ND-1000 spectrophotometer (Thermo Fisher Scientific, Waltham, MA, USA). The V3-V4 variable region of the 16S rRNA gene was amplified using primers 338F (5′-ACTCCGGGGAGGCAGCA3′) and 806R (5′-TCGGACTACHVGTWTCTAAT-3′). The resulting PCR product was purified and quantified using the Quant-iT PicoGreen dsDNA Assay Kit (Invitrogen, Carlsbad, CA, USA). Sequencing was conducted on the HiSeq 2500 PE 250 platform (Novogene Bioinformatics Technology Co., Ltd., Tianjin, China) after quantification.

The main tool used in the bioinformatics analysis was QIIME2. Venn diagrams were employed to depict shared and unique OTUs, thereby highlighting the similarities and differences among various treatments. The calculation of Alpha diversity indices, such as Shannon, Simpson, Chao1, and Ace, was performed at the ASV level using the ASV table in QIIME2, and these were represented through box plots. Beta diversity investigations used weighted UniFrac distance metrics to explore the structural changes in microbial communities across samples, visualized via principal coordinate analysis (PCoA) and non-metric multidimensional scaling (NMDS). The assessment of differences in the microbiota's relative abundance among treatments was done using LDA effect size (LEfSe) analyses and Random Forest analyses. The Spearman correlations between the representative bacteria at phylum, class, order, and family levels and the significant phenotype parameters concerning serum biochemical parameters, serum inflammatory factors, serum immunoglobulins, and short-chain fatty acid were plotted as heatmaps using the R package (version 2.15.3).

### 2.11 Statistical analysis

The quantitative analysis of the experimental data in this study was conducted utilizing the advanced statistical software IBM SPSS Statistics Version 27.0 (IBM Corp., Armonk, NY, USA). To determine statistical significance among treatment groups, a one-way analysis of variance (ANOVA) was implemented. Prior to conducting ANOVA, Levene's test for equality of variances was applied to ensure that the assumption of homogeneity of variance was satisfied across the groups. Following the initial ANOVA, *post hoc* analysis was performed using Tukey's Honest Significant Difference (HSD) test to conduct multiple pairwise comparisons and control the family-wise error rate. The threshold for statistical significance was established at *P* < 0.05. Additionally, instances where the *P*-value ranged from 0.05 to < 0.10 were interpreted as indicating a significant trend, warranting mention due to their potential biological relevance despite falling short of conventional levels of significance.

## 3 Results

### 3.1 Growth performance and nutrient apparent digestibility

As shown in Table 2, partial or totally substitute of soybean meal with a mixed meal (rapeseed meal, cotton meal, and sunflower meal) in the diet did not significantly influence the average daily gain (ADG), average daily feed intake (ADFI), or feed-to-gain ratio (F/G) of finishing pigs (*P* > 0.05).

**Table 2:** Effect of mixed meal replacement of soybean meal on the growth performance of finishing pigs^1^.

**Item**	**Treatments**			***p*-value**
	**CON**	**CSM**	**CMM**	
IBW, kg	97.52 ± 0.55	97.69 ± 0.54	97.58 ± 0.57	0.98
FBW, kg	126.16 ± 1.13	125.65 ± 1.36	125.14 ± 1.31	0.85
ADG, kg/d	1.10 ± 0.02	1.07 ± 0.04	1.06 ± 0.04	0.70
ADFI, kg/d	3.53 ± 0.09	3.46 ± 0.1	3.39 ± 0.05	0.53
F: G	3.21 ± 0.10	3.22 ± 0.06	3.21 ± 0.07	0.99

^1^Data are presented as mean ± SEM (*n* = 6). Within a row, means without a common superscript letter differ at *P* < 0.05. IBW, initial body weigh; FBW, final body weight; ADG, average daily gain; ADFI, average daily feed intake; F/G, ratio of feed to gain. CON, the control group was fed corn-soybean meal basal diet; CSM, the corn-soybean meal mixed meal group was fed with mixed meal to partially replace soybean meal in the basal diet; CMM, the corn mixed meal group was fed with mixed meal as a complete replacement of soybean meal in the basal diet.

Similarly, no significant effect was observed on the apparent digestibility of nutrients including crude protein, crude fat, and gross energy when soybean meal was replaced with a mixed meal (rapeseed meal, cotton meal, and sunflower meal) in the diet (*P* > 0.05) (Table 3).

**Table 3:** Effect of mixed meal replacement of soybean meal on apparent digestibility of nutrients of finishing pigs^1^.

**Item**	**Treatments**			***p*-value**
	**CON**	**CSM**	**CMM**	
Crude protein, %	73.42 ± 1.53	76.74 ± 1.22	73.30 ± 2.95	0.42
Crude fat, %	84.43 ± 1.05	85.73 ± 1.12	84.66 ± 1.74	0.43
Gross energy, %	85.80 ± 0.88	85.48 ± 0.75	83.60 ± 1.83	0.77

^1^Data are presented as mean ± SEM (*n* = 6). Within a row, means without a common superscript letter differ at *P* < 0.05. CON, the control group was fed corn-soybean meal basal diet; CSM, the corn-soybean meal mixed meal group was fed with mixed meal to partially replace soybean meal in the basal diet; CMM, the corn mixed meal group was fed with mixed meal as a complete replacement of soybean meal in the basal diet.

### 3.2 Serum inflammatory factors and immunoglobulins

It can be seen from the data in Table 4 that compared with the CON group, the serum IL-6 and IL-10 concentrations were significantly decreased in the CMM group (*P* < 0.05). However, there is no significant effect of mixed meal (rapeseed meal, cotton meal, and sunflower meal) replacing soybean meal in the diet on the serum IL-1β, IL-8, and TNF-α concentrations (*P* > 0.05).

**Table 4:** Effect of mixed meal replacement of soybean meal on serum inflammatory factors of finishing pigs^1^.

**Item**	**Treatments**			***p*-value**
	**CON**	**CSM**	**CMM**	
IL-1β, ng/L	24.81 ± 0.81	26.50 ± 2.49	29.69 ± 1.86	0.20
IL-6, ng/L	570.54 ± 30.53^a^	483.1 ± 32.72^ab^	456.14 ± 25.41^b^	0.04
IL-8, ng/L	260.48 ± 14.09	294.77 ± 30.56	281.96 ± 7.90	0.49
IL-10, ng/L	102.31 ± 5.50^a^	86.57 ± 5.89^ab^	81.72 ± 4.58^b^	0.04
TNF-α, ng/L	174.06 ± 12.43	182.69 ± 14.27	179.24 ± 6.97	0.87

^1^Data are presented as mean ± SEM (*n* = 6). Within a row, means without a common superscript letter differ at *P* < 0.05. CON, the control group was fed corn-soybean meal basal diet; CSM, the corn-soybean meal mixed meal group was fed with mixed meal to partially replace soybean meal in the basal diet; CMM, the corn mixed meal group was fed with mixed meal as a complete replacement of soybean meal in the basal diet.

Based on the findings in Table 5, our research indicates that substituting mixed meals (rapeseed meal, cotton meal, and sunflower meal) with soybean meal in the dietary does not have a significant effect on serum immunoglobulin concentrations, including IgA, IgG, and IgM (*P* > 0.05).

**Table 5:** Effect of mixed meal replacement of soybean meal on serum immunoglobulins of finishing pigs^1^.

**Item**	**Treatments**			***p*-value**
	**CON**	**CSM**	**CMM**	
IgA, μg/ml	21.14 ± 0.8	24.05 ± 1.12	22.01 ± 0.49	0.07
IgG, μg/ml	258.73 ± 15.31	274.69 ± 15.38	278.63 ± 10.02	0.57
IgM, μg/ml	31.41 ± 1.48	30.7 ± 1.57	31.22 ± 1.02	0.93

^1^Data are presented as mean ± SEM (*n* = 6). Within a row, means without a common superscript letter differ at *P* < 0.05. CON, the control group was fed corn-soybean meal basal diet; CSM, the corn-soybean meal mixed meal group was fed with mixed meal to partially replace soybean meal in the basal diet; CMM, the corn mixed meal group was fed with mixed meal as a complete replacement of soybean meal in the basal diet.

### 3.3 Antioxidant capacity

The results of the antioxidant capacity of serum are shown in Table 6. Our study showed that the replacement of soybean meal by mixed meal (rapeseed meal, cotton meal, and sunflower meal) in the diet did not significantly (*P* > 0.05) affect serum antioxidant indices, including total antioxidant capacity (T-AOC), catalase (CAT), and malondialdehyde (MDA) in finishing pigs.

**Table 6:** Effect of mixed meal replacement of soybean meal on antioxidant capacity of finishing pigs^1^.

**Item**	**Treatments**			***p*-value**
	**CON**	**CSM**	**CMM**	
T-AOC, U/ml	0.99 ± 0.11	1.03 ± 0.10	1.19 ± 0.16	0.49
CAT, U/ml	11.06 ± 0.68	11.75 ± 0.61	12.67 ± 0.51	0.25
MDA, nmol/ml	3.14 ± 0.36	2.78 ± 0.39	2.66 ± 0.20	0.61

^1^Data are presented as mean ± SEM (*n* = 6). Within a row, means without a common superscript letter differ at *P* < 0.05. T-AOC, total antioxidant capacity; CAT, catalase; MDA, malondialdehyde; CON, the control group was fed corn-soybean meal basal diet; CSM, the corn-soybean meal mixed meal group was fed with mixed meal to partially replace soybean meal in the basal diet; CMM, the corn mixed meal group was fed with mixed meal as a complete replacement of soybean meal in the basal diet.

### 3.4 Serum biochemical parameters

Table 7 shows that compared with the CON group, serum LDL-C levels were significantly lower in the CSM group (*P* < 0.05) and their T. BILI levels were significantly lower in the CMM group (*P* < 0.05). However, there is no significant effect of mixed meal (rapeseed meal, cotton meal, and sunflower meal) replacing soybean meal in the diet on the serum TP, ALT, CRE, AST, ALP, ALB, UREA, GLU, TG, CHO, and HDL-C levels (*P* > 0.05).

**Table 7:** Effect of mixed meal replacement of soybean meal on serum biochemical parameters of finishing pigs^1^.

**Item**	**Treatments**			***p*-value**
	**CON**	**CSM**	**CMM**	
TP, g/l	69.9 ± 2.30	71.45 ± 1.60	71.17 ± 1.06	0.80
ALT, U/L	46.66 ± 4.24	56.84 ± 5.24	46.84 ± 5.08	0.27
CRE, μmol/l	151.67 ± 1.91	159.59 ± 6.34	152.93 ± 5.49	0.50
AST, U/L	38.37 ± 1.98	41.65 ± 4.51	41.93 ± 3.57	0.73
ALP, U/L	125.61 ± 18.37	122.56 ± 2.47	99.84 ± 7.97	0.26
ALB, g/l	41.40 ± 2.56	41.18 ± 2.27	45.76 ± 5.63	0.64
UREA, mmol/l	3.44 ± 0.42	3.69 ± 0.39	3.27 ± 0.17	0.69
GLU, mmol/l	2.81 ± 0.12	2.77 ± 0.12	2.83 ± 0.07	0.93
TG, mmol/l	0.61 ± 0.03	0.62 ± 0.05	0.61 ± 0.04	0.97
CHO, mmol/l	10.84 ± 0.54	10.35 ± 0.5	9.96 ± 0.40	0.46
HDL-C, mmol/l	1.11 ± 0.06	1.07 ± 0.04	1.10 ± 0.04	0.85
LDL-C, mmol/l	1.81 ± 0.10^b^	2.32 ± 0.12^a^	2.16 ± 0.10^ab^	0.01
T. BILI, μmol/l	6.91 ± 0.66^b^	10.75 ± 1.45^ab^	12.67 ± 1.56^a^	0.02

^1^Data are presented as mean ± SEM (*n* = 6). Within a row, means without a common superscript letter differ at *P* < 0.05. TP, total protein; ALT, Glutathione transaminase CRE, Creatinine; AST, Glutathione aminotransferase; ALP, Alkaline phosphatase; ALB, Albumin; GLU, Glucose; TG, Triglycerides; CHO, Cholesterol; HDL-C, High-density lipoprotein; LDL-C, Low-density lipoprotein; T. BILI, Total bilirubin. CON, the control group was fed corn-soybean meal basal diet; CSM, the corn-soybean meal mixed meal group was fed with mixed meal to partially replace soybean meal in the basal diet; CMM, the corn mixed meal group was fed with mixed meal as a complete replacement of soybean meal in the basal diet.

### 3.5 Intestinal permeability and short-chain fatty acid contents

**Figure 1:**
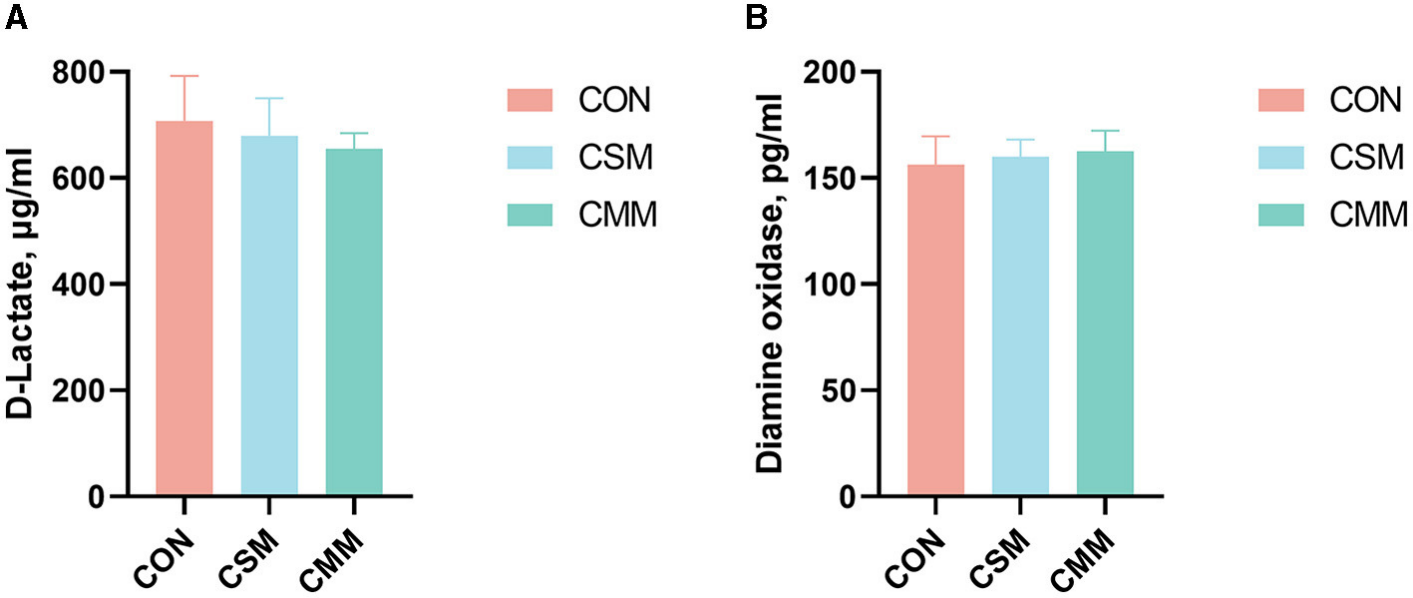
Effect of mixed meal replacement of soybean meal on intestinal permeability of finishing pigs. **(A)** D-lactate concentration in serum. **(B)** Diamine oxidase concentration in serum. CON, the control group was fed a corn-soybean meal basal diet; CSM, the corn-soybean meal mixed meal group was fed with a mixed meal to partially replace soybean meal in the basal diet; CMM, the corn mixed meal group was fed with a mixed meal as a complete replacement of soybean meal in the basal diet. Values are means ± SEM, *n* = 6. Labeled means in a row with different letters differ, *p* < 0.05.

From Figure 1, we can see that substituting mixed meals (rapeseed meal, cotton meal, and sunflower meal) with soybean meal in the dietary does not have a significant effect on serum D-lactate and DAO concentrations (*P* > 0.05).

As shown in Table 8, the replacement of soybean meal with a mixed meal (rapeseed meal, cotton meal, and sunflower meal) in the diet did not significantly influence the short-chain fatty acids (e.g., acetic, propionic, butyric, valeric, isobutyric, and isovaleric acids) in the colon contents (*P* > 0.05).

**Table 8:** Effect of mixed meal replacement of soybean meal on short-chain fatty acid content in the colon contents of finishing pigs^1^.

**Item**	**Treatments**			***p*-value**
	**CON**	**CSM**	**CMM**	
Acetic acid, mmol/g	16.33 ± 1.40	19.03 ± 2.28	18.66 ± 0.87	0.46
Propionic acid, mmol/g	12.93 ± 1.60	13.45 ± 1.52	13.32 ± 1.03	0.96
Butyric acid, mmol/g	5.87 ± 0.79	6.51 ± 0.85	6.74 ± 0.93	0.77
Valeric acid, mmol/g	1.43 ± 0.15	1.40 ± 0.17	1.94 ± 0.40	0.30
Isobutyric acid, mmol/g	2.75 ± 0.27	3.05 ± 0.25	3.09 ± 0.52	0.79
Isovaleric acid, mmol/g	3.64 ± 0.42	4.09 ± 0.59	4.56 ± 0.84	0.61

^1^Data are presented as mean ± SEM (*n* = 6). Within a row, means without a common superscript letter differ at *P* < 0.05. CON, the control group was fed corn-soybean meal basal diet; CSM, the corn-soybean meal mixed meal group was fed with mixed meal to partially replace soybean meal in the basal diet; CMM, the corn mixed meal group was fed with mixed meal as a complete replacement of soybean meal in the basal diet.

### 3.6 Gut microbiota composition and diversity

**Figure 2:**
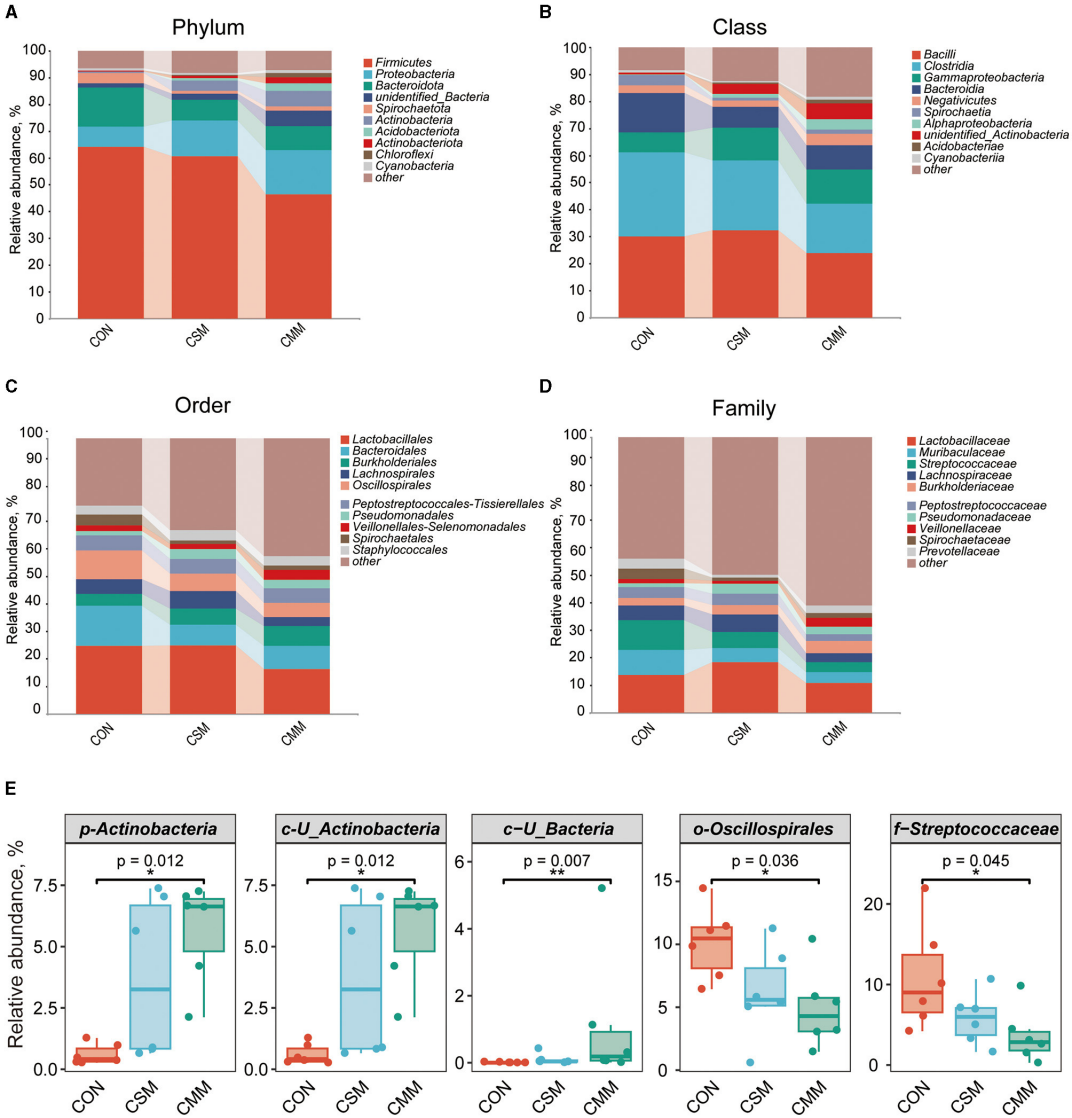
Effect of mixed meal replacement of soybean meal on Colonic microbial composition of finishing pigs. **(A)** Top 10 bacteria with phylum level abundance. **(B)** Top 10 bacteria with class level abundance. **(C)** Top 10 bacteria with order level abundance. **(D)** Top 10 bacteria with phylum family abundance. **(E)** Species with significant differences in the top 10 relative abundance at each level. Data was analyzed by Kruskal-Wallis ANOVA with Bonferroni posttest. CON, the control group was fed a corn-soybean meal basal diet; CSM, the corn-soybean meal mixed meal group was fed with a mixed meal to partially replace soybean meal in the basal diet; CMM, the corn mixed meal group was fed with a mixed meal as a complete replacement of soybean meal in the basal diet. Data are presented as mean ± SEM (*n* = 6). *Indicates significant difference at *p* < 0.05, **Indicates significant difference at *p* < 0.01.

At the phylum level (Figure 2A), the dominant bacteria were Firmicutes, Proteobacteria, and Bacteroidota. At the class level (Figure 2B), the dominant bacteria were *Bacilli, Clostridia*, and *Gammaproteobacteria*. At the order level (Figure 2C), the dominant bacteria were *Lactobacillales, Bacteroidales*, and *Burkholderiales*. At the family level (Figure 2D), the dominant bacteria were *Lactobacillaceae, Muribaculaceae*, and *Streptococcaceae*. From Figure 2E, we can see that compared with the CON group, the CMM group diet significantly increased the abundance of *Actinobacteria* at the phylum level (*P* < 0.05), *U_Actinobacteria* at the class level (*P* < 0.05), and *U_Bacteria* at the class level (*P* < 0.05). Figure 2E also showed that compared with the CON group, the CMM group diet significantly reduced the abundance of *Oscillospirales* at the order level (*P* < 0.05) and *Streptococcaceae* at the family level (*P* < 0.05).

**Figure 3:**
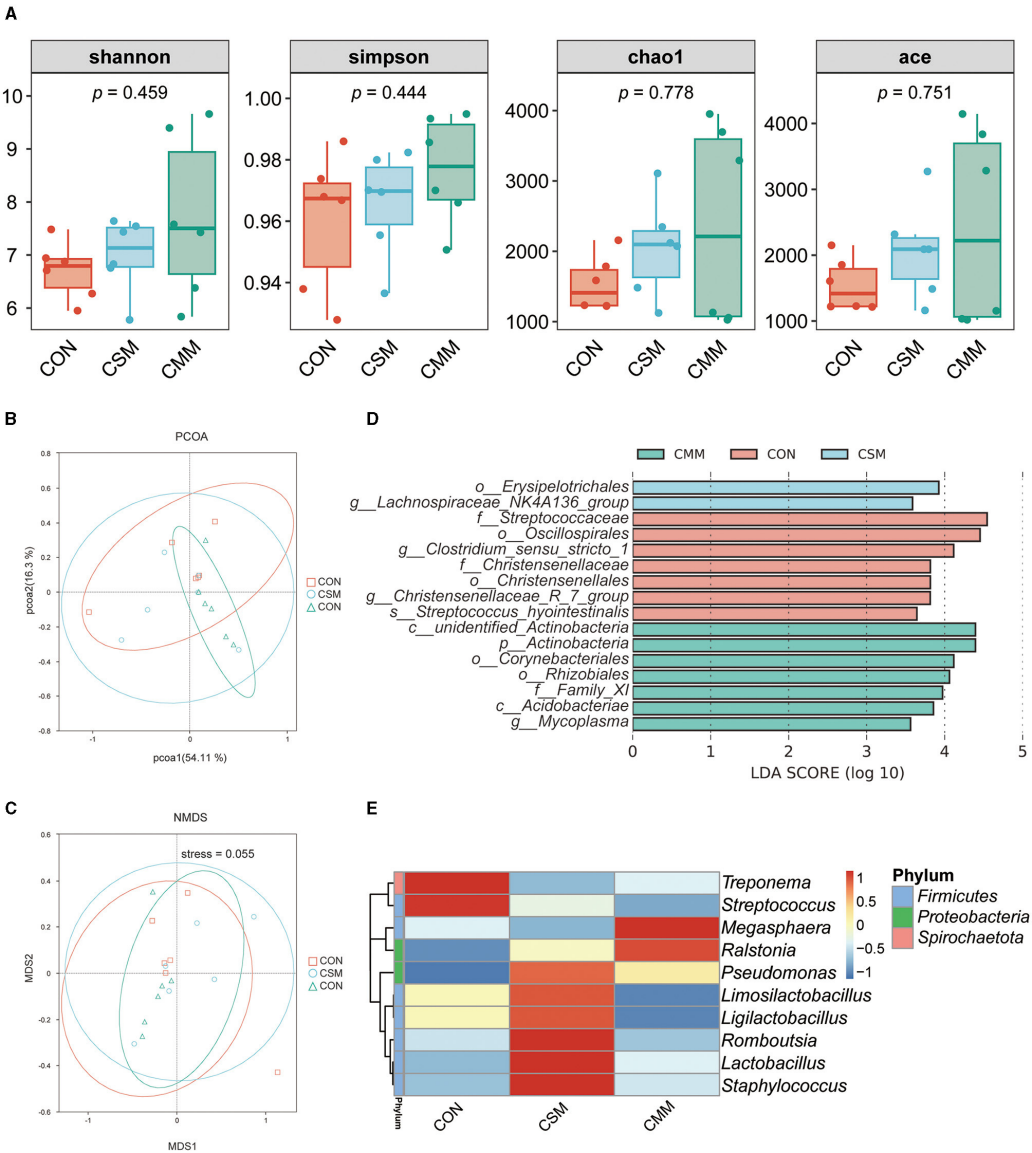
Effect of mixed meal replacement of soybean meal on colonic microbial diversity of finishing pigs. **(A)** Colon microbial alpha diversity metric (Shannon index, simpson index, chao1 index, ace index) of the three groups. **(B)** The principal coordinate analysis (PCoA) based on weighted_unifrac algorithm. **(C)** Non-Metric Multidimensional Scaling (NMDS) based on weighted_unifrac algorithm. **(D)** The linear discriminant analysis effect size (LEfSe) analysis (LDA score > 3.5) identified the biomarker bacterial species in the three groups. **(E)** Cluster heat map of the top 10 genera in terms of abundance. CON, the control group was fed corn-soybean meal basal diet; CSM, the corn-soybean meal mixed meal group was fed with mixed meal to partially replace soybean meal in the basal diet; CMM, the corn mixed meal group was fed with mixed meal as a complete replacement of soybean meal in the basal diet.

As shown in Figure 3A, the replacement of soybean meal with a miscellaneous meal in the diet did not significantly impact the alpha diversity of colon microbiota, as indicated Shannon index, Simpson index, Chao1 index, and Ace index (*P* > 0.05).

In terms of beta diversity, the distributional distances between the three groups (CON, CSM, and CMM) were not distinctly separated, as evidenced by the results of the PCoA (Figure 3B) and NMDS (Figure 3C) analyses. LEfSe analysis (Figure 3D) was used to identify bacteria that were not significantly different between the three groups (CON, CSM, and CMM). The LEfSe analysis indicated that seven bacteria including *Streptococcaceae* (Family), *Oscillospirales* (Order), *Clostridium_sensu_stricto_1* (Genes), *Christensenellaceae* (Family), *Christensenellales* (Order), *Christensenellaceae_R_7_group* (Genes), *Streptococcus_hyointestinalis* (Species) were enriched in CON group. However, the CMM group enriched two bacteria including *Erysipelotrichales* (Order) and *Lachnospiraceae_NK4A136_group* (Genes). The CSM group enriched seven bacteria including *unidentified_Actinobacteria* (Class), *Actinobacteria* (Phylum), *Corynebacteriales* (Order), *Rhizobiales* (Order), *Family_XI* (Family), *Acidobacteriae* (Class), and *Mycoplasma* (Genes). As depicted in Figure 3E, we identified the top 10 genera in terms of abundance.

**Figure 4:**
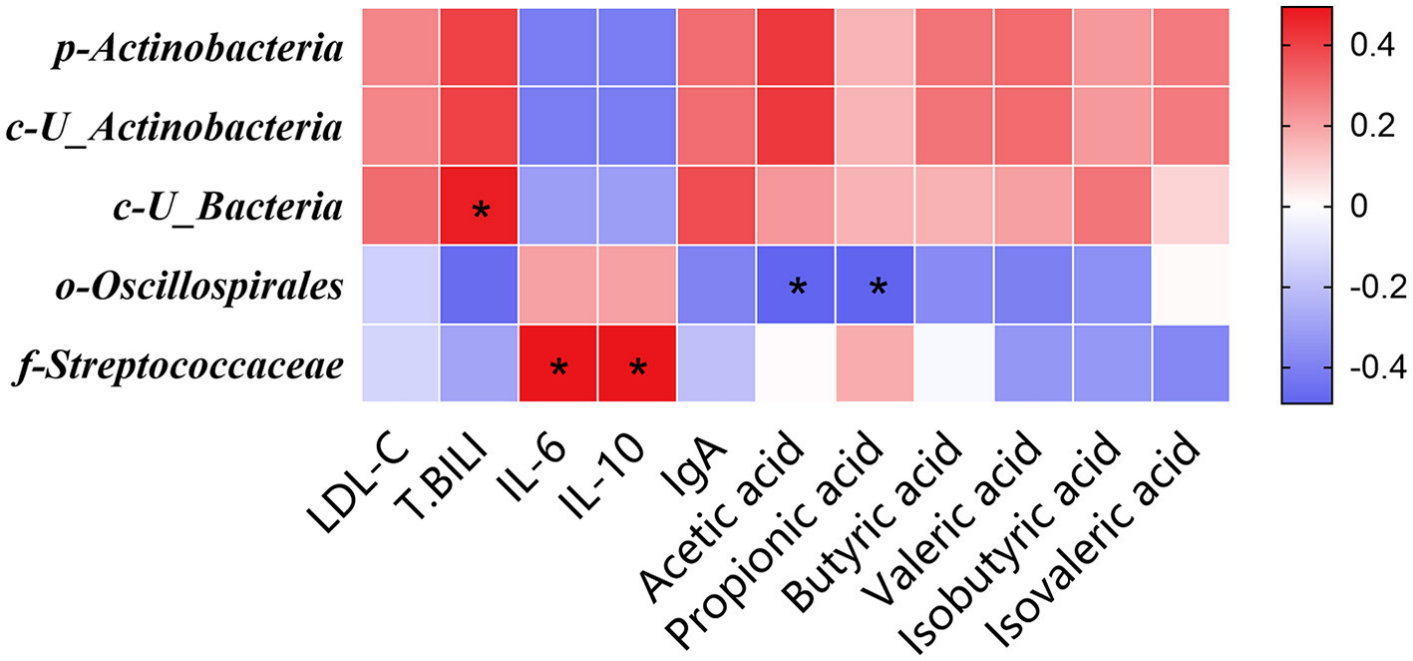
The Spearman correlation analysis of gut microbial composition at phylum, class, order, and family with serum biochemical parameters, serum inflammatory factors, serum immunoglobulins, short-chain fatty acid in finishing pigs. Spearman correlation coefficients of LDL-C, T. BILI, IL-6, IL-10, IgA, acetic acid, propionic acid, butyric acid, valeric acid, isobutyric acid, and isovaleric acid at phylum, class, order, and family level, are represented by color ranging from red (positive correlation) to blue (negative correlation). *Indicates statistically significant difference (*P* < 0.05). LDL-C, Low-density lipoprotein; T. BILI, Total bilirubin; IL-6, interleukin 6; IL-10, interleukin 10; IgA, immunoglobulin A.

The Spearman correlation analysis depicted in Figure 4 revealed a lack of statistically significant association between the relative abundance of the phylum Actinobacteria and the salient phenotypic manifestations resulting from the substitution of soybean meal with the mixed meal (rapeseed meal, cotton meal, and sunflower meal) in finishing pigs (*P* > 0.05). This absence of significant correlation persisted at the class level with *U_Actinobacteria* (*P* > 0.05). In contrast, a statistically significant positive correlation was identified at the class level between the relative abundance of *U_Bacteria* and the serum T. BILI concentrations (*P* < 0.05). Moreover, a significant negative correlation was detected at the order level between the relative abundance of *Oscillospirales* and the levels of acetic and propionic acids in the colonic contents (*P* < 0.05). Additionally, there was a significant positive correlation between the serum concentrations of IL-6 and IL-10 and the relative abundance of the family *Streptococcaceae* (*P* < 0.05).

## 4 Discussion

Feed costs significantly influence the profitability of pork farmers, representing a substantial portion of the overall pig-rearing expenses. Globally, soybean meal (SBM) is a prevalent protein source; nonetheless, escalating the SBM quantity in pig diets subsequently increases feed costs (10, 11). Rapeseed meal is a by-product of oil extraction from rapeseed. Rapeseed meal is an important alternative to soybean meal as a feed protein ingredient and is easily digested and utilized by animals. Rapeseed meal contains a complete range of amino acids (12). Similarly, cottonseed meal, derived from cottonseed post-crushing and oil leaching, and sunflower seed meal, obtained from partially hulled sunflower seeds via pre-pressure leaching or direct solvent leaching for oil, are valuable by-products. Rapeseed meal, cottonseed meal, and sunflower seed meal have traditionally been explored as substitutes for soybean meal in livestock and poultry feed, as documented in previous studies (13–15). However, there is a dearth of research exploring the implications of these alternatives (rapeseed meal, cottonseed meal, and sunflower seed meal) on growth performance, nutrient apparent digestibility, serum immunoglobulins, serum antioxidant capacity, intestinal permeability, short-chain fatty acid content, and diversity of gut microbiota in finishing pigs. Therefore, our study aims to illuminate the effects of replacing SBM with rapeseed meal, cottonseed meal, and sunflower seed meal in the diets of finishing pigs.

As expected, the present results showed that partial or complete replacement of soybean meal by miscellaneous meal does not affect the growth performance and development rate in finishing pigs. The findings of our study are consistent with previous research, demonstrating that the replacement of 11% of soybean meal in control diets with 13% double-low rapeseed meal had no adverse effects on the growth performance of growing pigs weighing 62 kg (16). Similar studies have also indicated that the growth performance of growing pigs is not significantly influenced by the partial substitution of 5% soybean meal with rapeseed meal and cottonseed meal in their diet (4). Furthermore, it has been shown that the inclusion of sunflower seed meal at 5%, 10%, and 15% in the ration as a replacement for soybean meal does not have a significant impact on the growth performance of growing pigs weighing 62 kg (17). The study showed that there were no differences in sow BW changes during gestation, in sow BW on day 1 post-farrowing, or at weaning due to dietary treatments, and there were no differences in ADFI between gestation and lactation diets (18). In line with these findings, another study observed no differences in body weight and daily weight gain among growing-finishing pigs weighing 29.94 ± 0.06 kg when fed diets containing rapeseed meal. Thus, our experimental results corroborate the aforementioned correlations between feed composition and growth performance outcomes. But from week 13 of the growing season onward, increasing dietary rapeseed additions lowered body weight and weight per increase (19), this could be attributed to the elevated levels of rapeseed meal, which led to an increase in the fiber content of the feed (10). The inclusion of rapeseed in the diets of monogastric animals has been somewhat restricted due to its substantial fiber and oligosaccharide content, which stands at about 2.5% (20). Consistent with the literature, a significant study by Hansen et al. (21) revealed a marked decrease in both average daily gain and feed conversion ratios, indicating a potential negative impact on the feed intake of growing-finishing pigs. This effect was observed when the animals were fed nutritionally balanced diets that contained escalating levels of rapeseed meal. Additionally, throughout the study, G/F ratios tended to fall as dietary rapeseed addition increased. It has been found that the gradual addition of rapeseed meal to feed in the range of 0–30% will linearly reduce the ADFI of growing pigs, and there is a tendency to linearly reduce ADG (22). Hong et al. (23) also found that ADG overall responded quadratically to the rising dietary level of rapeseed meal, increasing by 17% when the dietary level of rapeseed meal was increased from 0% to 20% and decreasing by 16% when the dietary level of rapeseed meal was increased from 20% to 40%. The divergence in these results might be attributed to the varying proportions of soybean meal, rapeseed meal, cottonseed meal, and sunflower meal in the pig feed, as well as the differing content of anti-nutritional factors in these meals.

Nutrient apparent digestibility serves as an indicator of the animal's capacity to digest and absorb the nutrients present in their feed. The role of anti-nutritional factors (ANFs) is indeed an important consideration when substituting soybean meal with the mixed meal in pig diets. Previous studies have indicated that ANFs can hinder nutrient absorption and affect digestibility in pigs (24–26). Our experimental findings suggest that replacing the soybean meal with the mixed meal does not significantly impact the apparent nutrient digestibility in finishing pigs. Consequently, we did not calculate the anti-nutritional factors of the mixed meal in our experiment. A stronger absorption ability is typically associated with more favorable animal growth. Shim et al. (4) found that the replacement of rapeseed meal and cottonseed meal with soybean meal in different proportions at different stages did not significantly alter nutrient apparent digestibility in growing pigs, including crude protein, crude fat, and gross energy, this is consistent with the results of our experiments. Similarly, it has been found that the replacement of soybean meal with rapeseed meal (380 g/kg replacement rate during the growing period and 720 g/kg replacement rate during the finishing period) in the ration resulted in a decrease in crude protein apparent digestibility but no effect on crude fat apparent digestibility in finishing pigs (27). However, the substitution of soybean meal with different varieties of sunflower seed meal revealed varying effects on the apparent digestibility of gross energy in growing pigs (3), this discrepancy may be attributed to the fact that different processing conditions, such as temperature, pressure, or duration, can alter the chemical composition and consequently the energy content of sunflower seed meal (28). These findings suggest that the substitution of soybean meal with rapeseed meal, cottonseed meal, and sunflower seed meal does not significantly affect the growth performance and apparent nutrient digestibility in growing pigs.

Proinflammatory cytokine response to immune challenge is an important criterion for the degree of cellular immunity. The behavioral, neuroendocrine, and metabolic effects of three cytokines, namely tumor necrosis factor-alpha (TNF-α), interleukin-1 (IL-1), and interleukin-6 (IL-6), are significant (29). The excessive production of cytokines, including IL-1β, leads to the diversion of nutrients from growth processes to support the immune system (30). In our study, compared with the CON group, the serum IL-6 and IL-10 concentrations were significantly decreased in the CMM group. However, there is no significant effect of mixed meal replacing soybean meal in the diet on the serum IL-1β, IL-8, and TNF-α concentrations. Consistent with previous studies, our trial also observed that the substitution of canola meal for soybean meal did not result in significant changes in blood levels of TNF-α in piglets (7 kg) (23). In contrast to the aforementioned findings, results revealed that the inclusion of 8% fermented canola meal and feed additives in the diet did not lead to significant differences in IL-6 levels compared to the control group. However, in the group fed with 8% fermented canola meal but without feed additives, there was a notable increase in IL-6 levels (31). Furthermore, a previous study also demonstrated that feeding piglets diets containing either 8% or 6% fermented canola meal resulted in significant reductions in plasma concentrations of IL-6 compared to the control group (32). This observation suggests that the fermented components present in canola meal may have a stimulating effect on the production of IL-6.

The serum IgA, IgG, and IgM are serum antibodies and major components of humoral immunity in pigs. Measurement of immunoglobulin levels in the blood can be used to assess the effect of feed on the immune status of the animal. The findings of the previous study indicate that the supplementation of fermented rapeseed feed and other feed additives in the group (28 d) resulted in significant increases in immunoglobulin levels in the blood of pigs. Specifically, compared to the control group, the blood levels of IgG, IgA, and IgM were elevated by 42.7%, 75.7%, and 44.1%, respectively (31). Moreover, the inclusion of fermented canola meal in the diets of pigs (28 d) led to notable increases in plasma concentrations of IgG and IgA. These results suggest that the incorporation of fermented rapeseed feed stimulates the immune system and enhances the synthesis of immunoglobulins in pigs (32). The study findings revealed that the inclusion of rapeseed meal in the diet did not lead to significant alterations in serum concentrations of IgA, IgG, and IgM in pigs after a 7-day breeding period (23). The findings of our study suggest that the substitution of soybean meal with mixed meal did not yield statistically significant changes in the blood levels of IgA, IgG, and IgM in finishing pigs. However, it is worth noting that this outcome may be influenced by factors such as the dosage of mixed meal incorporated and whether or not it underwent fermentation.

The quantification of antioxidant biomarkers serves as an indicative measure of the porcine health status. Enhanced antioxidant potential mitigates inflammatory processes and augments the functionality of the immune defenses, consequently bolstering the swine's resilience against pathogenic challenges. Our research indicated that the substitution of soybean meal with a mixed meal formulation did not significantly alter the antioxidant profiles, specifically T-AOC, CAT, and MDA, in finishing pigs. This outcome implies that the mixed meal does not detrimentally impact the systemic antioxidative capabilities of the finishing pigs during their growth phase. Previous research has demonstrated that substituting soybean meal with fermented canola meal leads to a significant elevation in serum CAT activity and a notable reduction in MDA levels (*P* < 0.05) in growing pigs (31). Additionally, there is evidence suggesting that fermented rapeseed meal extracts are capable of mitigating oxidative stress (33). The marked divergence between the outcomes of these studies and our own may stem from the fact that the mixed meal utilized in our experimental design was not subjected to a fermentation process.

Serum biochemical parameters are generally used to reflect the physiological and health status of animals. According to our findings, compared with the CON group, serum LDL-C levels were significantly lower in the CSM group and their T. BILI levels were significantly lower in the CMM group. Low-density lipoprotein (LDL) is a crucial blood lipid responsible for the transportation of cholesterol and fats from the liver to various tissues within the body (34). However, feeding diets containing fermented rapeseed meal had no significant effect on the concentration of LDL in the plasma of piglets (32), this may be related to the different growth stages of pigs. Derived from heme metabolism, bilirubin is a tetrapyrrole compound that exhibits diverse functions, encompassing cell signaling, metabolic regulation, and immunomodulation. These multifaceted roles of bilirubin hold significant clinical and therapeutic implications (35). In contrast to our experimental results, the previous findings demonstrated a significant increase in plasma bilirubin levels among piglets supplemented with fermented rapeseed feed and other feed additives (31).

The preservation of an intact intestinal mucosal barrier is essential for protecting against pathogenic bacteria (36, 37). When the intestinal mucosal barrier is compromised, it can result in elevated intestinal permeability. Several factors, such as D-lactate concentration and DAO activity in serum, contribute to the functionality of the intestinal barrier (38–40). These factors have been identified as markers for evaluating the degree of intestinal mucosal damage and repair (41). D-lactate, a metabolic byproduct of intestinal bacteria, is typically absent in mammals due to the lack of D-lactate dehydrogenase. Consequently, healthy individuals maintain low levels of D-lactate (42). However, when the integrity of the intestinal mucosa is compromised, a substantial release of D-lactate into the bloodstream occurs, indicating the status of intestinal mucosal integrity and maturity (43). DAO, an enzyme catalyzed by deaminases, is exclusively found in the villi of the upper small intestine. Elevated levels of DAO serve as an indicator of increased intestinal epithelial permeability or damage to the intestinal barrier function (44, 45). In the present study, substituting mixed meals with soybean meal in the dietary does not have a significant effect on serum D-lactate and DAO concentrations. The findings suggest that the substitution of soybean meal with a mixed meal does not compromise the integrity of the intestinal barrier. Short-chain fatty acid was reported to enhance gut barrier function and modulate gut immune response (46). Some experiments demonstrated an increase in short-chain fatty acids in the colon of pigs following the treatment of canola meal with cellulase, two pectinases, or alkaline treatment (47, 48).

Intestinal flora and its metabolites play an important role in animal health, and diet is one of the main factors influencing the composition of intestinal flora. It is known from previous studies that Firmicutes and Bacteroidetes are the two most dominant bacterial phylum in this race of pigs (49). Notably, a diet supplemented with 20% rapeseed meal considerably reduced the relative abundance of the Bacteroidetes phylum while tending to increase the relative abundance of the Firmicutes phylum (23). Consistently, our results showed that Firmicutes is the most dominant phylum in finishing pigs' colon. Furthermore, the analysis of microbial alpha diversity revealed that the utilization of miscellaneous meals as a complete replacement for soybean meal did not have any impact on either Chao1 or Simpson's index, these findings are consistent with Gu et al. (14). Other studies also showed that a similar Shannon index, observed OTUs, Chao1, and Phylogenetic diversity in whole trees in the colon of growing pigs (25 ± 2 kg) using 100% replacement of soybean meal with rapeseed meal (50). Although the increased bacterial load from the small intestine to the large intestine resulted in a difference in intestinal location from expected, the diversity was similar between dietary groups at each location. It is important to note that research has shown that feeding fermented soybean meal to growing pigs (17.46 ± 1.97 kg) instead of regular soybean meal greatly decreased the amount of Escherichia coli in the colon while dramatically increasing the amount of Lactobacillus (51). In a similar vein, it was also found that the addition of fermented soybean meal instead of soybean meal could significantly increase the number of lactic acid bacteria in the feces of piglets (7 kg), while concurrently reducing the total count of Coliforms and Clostridium perfringens (52). This outcome is attributable to the fermentation of rapeseed meal. However, there is currently little research on the intestinal flora of developing pigs fed cottonseed meal and sunflower meal rather than soybean meal, necessitating more investigation. In summary, our experimental results showed no significant difference in bacterial flora, which suggests that the replacement of soybean meal with miscellaneous mixed meals (rapeseed meal, cottonseed meal, and sunflower meal) did not affect the colonic intestinal environment of finishing pigs.

## 5 Conclusions

Collectively, this study's findings indicate that the integration of mixed meal (rapeseed meal, cotton meal, and sunflower meal) as a substitute for soybean meal in the diet did not significantly negatively impact the growth performance, nutrient apparent digestibility, serum immunoglobulins, serum antioxidant capacity, intestinal permeability, short-chain fatty acid content, and diversity of gut microbiota in finishing pigs. Therefore, these findings suggest that miscellaneous meals (rapeseed meal, cottonseed meal, and sunflower seed meal) serve as a partial or complete substitute for soybean meal in the diets of finishing pigs, potentially offering excellent alternative protein sources.

## Data availability statement

The original contributions presented in the study are publicly available. This data can be found here: https://www.ncbi.nlm.nih.gov/; PRJNA1030319.

## Ethics statement

The animal study was approved by Animal Care and Use Committee of the Guangdong Academy of Agricultural Sciences. The study was conducted in accordance with the local legislation and institutional requirements.

## Author contributions

ZH: Investigation, Formal Analysis, Visualization, Writing—original draft. SL: Investigation, Formal Analysis, Visualization, Writing—original draft. XW: Formal Analysis, Validation, Writing—original draft, Data curation. SC-a: Methodology, Data curation, Writing—review & editing. XZ: Methodology, Validation, Data curation, Writing—review & editing. LH: Methodology, Validation, Data curation, Writing—review & editing. YL: Validation, Data curation, Writing—review & editing. SC-b: Validation, Data curation, Writing—review & editing. HZ: Validation, Data curation, Writing—review & editing. DD: Validation, Data curation, Writing—review & editing. KG: Methodology, Resources, Writing—review & editing. XY: Methodology, Resources, Writing—review & editing. ZJ: Project administration, Conceptualization, Funding acquisition, Writing—review & editing, Supervision. LW: Project administration, Conceptualization, Funding acquisition, Writing—review & editing, Supervision.
